# Complete Genome Sequence of a Potential Probiotic, *Lactobacillus pentosus* MP-10, Isolated from Fermented Aloreña Table Olives

**DOI:** 10.1128/genomeA.00854-16

**Published:** 2016-09-15

**Authors:** Hikmate Abriouel, Beatriz Pérez Montoro, María del Carmen Casado Muñoz, Leyre Lavilla Lerma, Marina Hidalgo Pestaña, Natacha Caballero Gómez, Charles M. A. P. Franz, Antonio Gálvez, Nabil Benomar

**Affiliations:** a Área de Microbiología, Departamento de Ciencias de la Salud, Facultad de Ciencias Experimentales, Universidad de Jaén, Jaén, Spain; b Universite de Strasbourg, Faculte de Pharmacie, Illkirch Eq. Chimie Analytique MBA, Strasbourg, France; c Department of Microbiology and Biotechnology, Max Rubner-Institut, Kiel, Germany

## Abstract

We report here a 3,698,214-bp complete genome sequence of a potential probiotic *Lactobacillus pentosus* strain, MP-10, isolated from brines of naturally fermented Aloreña green table olives; it is considered the largest sequenced genome among lactobacilli to date. The annotated genome sequence revealed the presence of 3,558 open reading frames (ORFs) and 87 structural RNAs.

## GENOME ANNOUNCEMENT

Lactobacilli are ubiquitous in the environment (1, 2), and they have been used in food fermentation and as probiotics (3). Generally, probiotic lactobacilli were traditionally isolated from human sources (milk and intestinal tract). However, a search for probiotic lactic acid bacteria (LAB) from nondairy origin, such as fruits and vegetables, has increased in the last years (4-6). Naturally fermented Aloreña green table olives are considered a natural source of potential probiotics, especially *Lactobacillus pentosus* strains (7, 8).

The genomic DNA of a potential probiotic, *L. pentosus* MP-10, which was isolated from brines of naturally fermented Aloreña green table olives (7), was obtained using the PureGene core kit B, according to the manufacturer’s instructions (Qiagen, Spain). Genome sequencing, assembly, and annotation were done at Lifesequencing *S.L*. (Valencia, Spain). The resulting reads were assembled *de novo* using the Hierarchical Genome Assembly Process (HGAP3.0) approach (SMRT analysis version: 2.3.0, patch #4) with default parameters and with the minimum seed read length set at 6,000 bp.

The first draft genome sequenced was obtained in 2011 (EMBL accession numbers FR871759 to FR871848) by pyrosequencing technology (GS FLX Titanium system; 454 Life Sciences) and revealed the presence of a circular 3,835,873-bp chromosome (108 contigs) and three plasmids (18 to 53 kb) (7). However, resequencing of the *L. pentosus* MP-10 genome by using PacBio RS II technology revealed that the assembly contained six contigs (one chromosome and five plasmids). Further analysis aiming to circularize the contigs derived from the assemblies was done using the publicly available tool Circlator based on the algorithm reported by Hunt et al. (9). The assembled genome sequences were annotated using the Prokka annotation pipeline, version 1.11 (10–13). The single circular chromosome consisted of 3,698,214 bp, with an estimated mol% G+C content of 46.32% and around 250× coverage. The five plasmids ranged between 29 and 56 kb in size: pLPE-1 (29,077 bp; mol% G+C content, 40.77%), pLPE-2 (34,764 bp; mol% G+C content, 39.93%), pLPE-3 (38,717 bp; mol% G+C content, 42.50%), pLPE-4 (43,946 bp; mol% G+C content, 40.09%), and pLPE-5 (46,498 bp; mol% G+C content, 39.52%). The complete and newly annotated genome sequence revealed the presence of 3,558 open reading frames (ORFs) (2,971 canonical and 587 noncanonical) and 87 structural RNAs (sRNAs) (16 rRNA and 71 tRNA). The genome sequence of *L. pentosus* MP-10 can be considered the currently largest genome among lactobacilli known to date, which may reflect the ecological flexibility of this bacterium via metabolic diversity and lifestyle adaptability as a result of bacterial evolution (gene duplication and horizontal gene transfer [HGT]). We also identified the presence of two clustered regularly interspaced short palindromic repeat (CRISPR) clusters (types I and II) that represent an acquired “immune system,” providing protection against mobile genetic elements (viruses, transposable elements, and conjugative plasmids) (14). The availability of the complete genome sequence will aid in future investigations into the probiotic properties of the *L. pentosus* MP-10 strain.

### Accession number(s).

The whole-genome sequence of *L. pentosus* MP-10 has been deposited at the EMBL Nucleotide Sequence Database under accession numbers FLYG01000001 to FLYG01000006.
